# Supplementary figures and images for: Differential MicroRNA Expression in Porcine Endometrium Related to Spontaneous Embryo Loss during Early Pregnancy
Source: Int J Mol Sci. 2022 Jul 24;23(15):8157. doi: 10.3390/ijms23158157 (PMC9331794; doi:10.3390/ijms23158157)

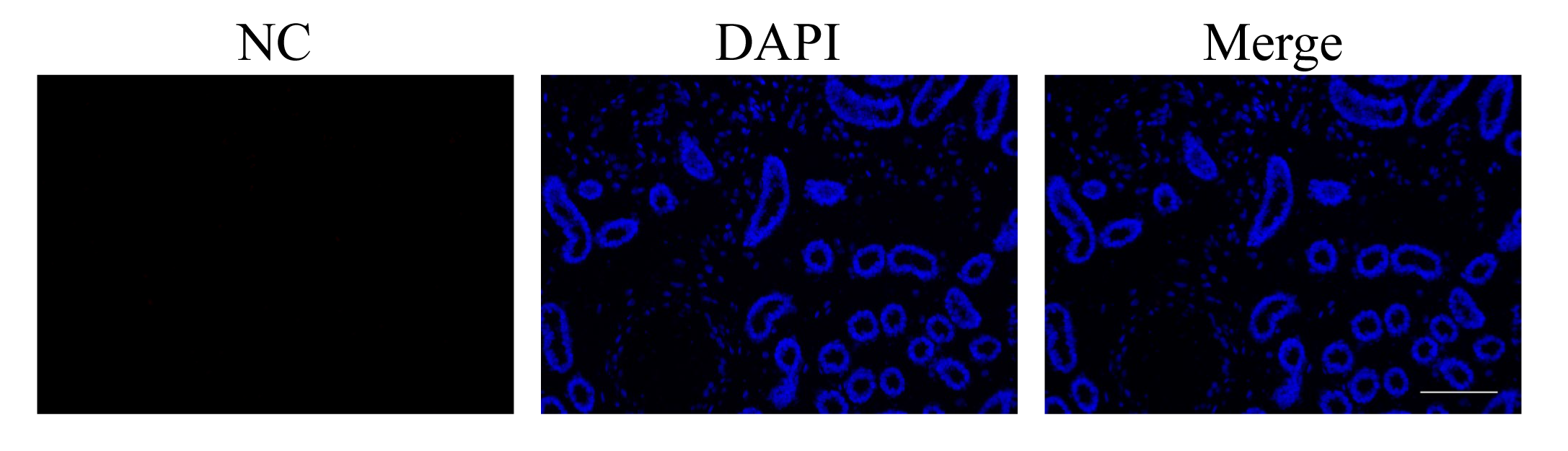

Supplement: Supplementary file 1 [file ijms-23-08157-s001.zip › Supplement Figure S1.tif]
